# Resonant Sand

**Published:** 1881-10

**Authors:** 


### RESONANT SAND.

M. Lenz, in a recent communication to the Geographical Society of France in regard to his voyage to Timbuctoo, speaks of a curious phenomenon that he witnessed, and which he calls "resonant sand."

"In the Inguidi," says he, "a region of sand dunes very difficult to cross, I observed a phenomenon which was as rare as it was interesting—resonant or musical sand. All at once one hears in the desert, issuing from a sand dune, a prolonged, smothered sound quite like the noise of a trumpet. It lasts for some seconds, and then stops to resume itself in another direction. The phenomenon renders the traveler anxious. I suppose it proceeds from the friction against one another of the burning hot grains of quartz, which are simply laid one over the other and are always in motion.—*Revue Scientifique.*

